# SpacePHARER: sensitive identification of phages from CRISPR spacers in prokaryotic hosts

**DOI:** 10.1093/bioinformatics/btab222

**Published:** 2021-04-01

**Authors:** Ruoshi Zhang, Milot Mirdita, Eli Levy Karin, Clovis Norroy, Clovis Galiez, Johannes Söding

**Affiliations:** Quantitative and Computational Biology, Max Planck Institute for Biophysical Chemistry, Göttingen, Germany; Quantitative and Computational Biology, Max Planck Institute for Biophysical Chemistry, Göttingen, Germany; Quantitative and Computational Biology, Max Planck Institute for Biophysical Chemistry, Göttingen, Germany; Quantitative and Computational Biology, Max Planck Institute for Biophysical Chemistry, Göttingen, Germany; Quantitative and Computational Biology, Max Planck Institute for Biophysical Chemistry, Göttingen, Germany; University of Grenoble Alpes, CNRS, Grenoble INP/Institute of Engineering, LJK, Grenoble, France; Quantitative and Computational Biology, Max Planck Institute for Biophysical Chemistry, Göttingen, Germany; Campus-Institut Data Science (CIDAS), Göttingen, Germany

## Abstract

**Summary:**

SpacePHARER (CRISPR Spacer Phage–Host Pair Finder) is a sensitive and fast tool for *de novo* prediction of phage–host relationships via identifying phage genomes that match CRISPR spacers in genomic or metagenomic data. SpacePHARER gains sensitivity by comparing spacers and phages at the protein level, optimizing its scores for matching very short sequences, and combining evidence from multiple matches, while controlling for false positives. We demonstrate SpacePHARER by searching a comprehensive spacer list against all complete phage genomes.

**Availability and implementation:**

SpacePHARER is available as an open-source (GPLv3), user-friendly command-line software for Linux and macOS: https://github.com/soedinglab/spacepharer.

**Supplementary information:**

Supplementary data are available at *Bioinformatics* online.

## 1 Introduction

Viruses of bacteria and archaea (phages) are the most abundant biological entities in nature. However, little is known about their roles in the microbial ecosystem and how they interact with their hosts, as cultivating most phages and hosts in the lab is challenging. Many prokaryotes (40% of bacteria and 81% of archaea) possess an adaptive immune system against phages, the Clustered Regularly Interspaced Short Palindromic Repeat (CRISPR)-CRISPR associated (Cas) system (Burstein *et al.*, 2016). After surviving a phage infection, they can incorporate a short DNA fragment (28–42 nt) as a spacer in a CRISPR array. The transcribed spacer will be used with other Cas components for a targeted destruction of future invaders. Some CRISPR-Cas systems require a 2–6 nucleotide long, highly conserved protospacer-adjacent motif (PAM) flanking the viral target to prevent autoimmunity. Multiple spacers targeting the same invader are not uncommon, due to either multiple infection events or the primed spacer acquisition mechanism identified in some CRISPR subtypes. CRISPR spacers have been previously exploited to identify phage–host relationships (Biswas *et al.*, 2013; Dion *et al.*, 2021; Paez-Espino *et al.*, 2016; Shmakov *et al.*, 2017; Stern *et al.*, 2012). These methods compare individual CRISPR spacers with phage genomes using BLASTN (Altschul *et al.*, 1990) and apply stringent filtering criteria, e.g. allowing only up to two mismatches. They are thus limited to identifying very close matches. However, a higher sensitivity is crucial because phage reference databases are very incomplete and often will not contain phages highly similar to those to be identified. To increase sensitivity, (i) we compare protein coding sequences because phage genomes are mostly coding, and, to evade the CRISPR immune response, are under pressure to mutate their genome with minimal changes to the amino acids; (ii) we choose an optimized substitution matrix and gap penalties for short, highly similar proteins; and (iii) we combine evidence from multiple spacers matching to the same phage genome.

## 2 Materials and methods

*Input.* SpacePHARER accepts spacer sequences as multiple FASTA files each containing spacers from a single prokaryotic genome or as multiple output files from the CRISPR detection tools PILER-CR (Edgar, 2007), CRT (Bland *et al.*, 2007), MinCED (Skennerton, 2016) or CRISPRDetect (Biswas *et al.*, 2016). Phage genomes are supplied as separate FASTA files or can be downloaded by SpacePHARER from NCBI GenBank (Benson *et al.*, 2013). Optionally, additional taxonomic labels can be provided for spacers or phages to be included in the final report.

**Fig. 1.**
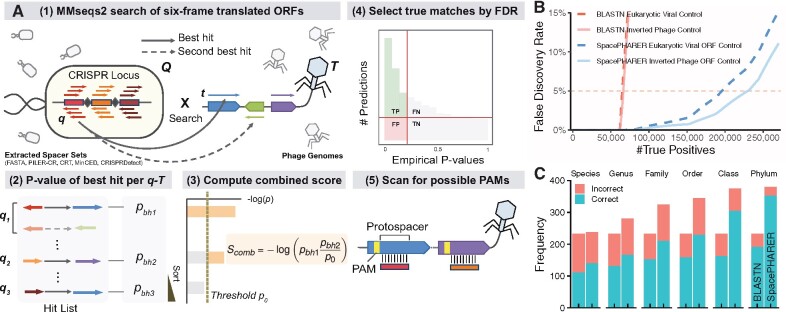
(**A**) SpacePHARER algorithm. A query set *Q* consists of 6-frame translated ORFs (*q*) from CRISPR spacers, and a target set *T* consists of 6-frame translated ORFs (*t*) of phage proteins. (1) Search all *q*s against all *t*s using MMseqs2. Align the *q—t* hits on nucleotide level and prioritize near-perfect nucleotide hits. (2) For each *q—T* pair, compute the *P*-value for the best hit from first-order statistics. (3) Compute score $S_{\text{comb}}$ by combining the best-hit *P*-values from multiple hits between *Q* and *T* using a modified truncated-product method. (4) Estimate the FDR by searching a null database. (5) Scan for possible protospacer adjacent motif (PAM). (**B**) Performance comparison between SpacePHARER (blue) and BLASTN (red) using inverted phage sequences (solid lines) or eukaryotic viral ORFs as null set (dashed lines) demonstrated by expected number of true positive (TP) predictions at different false discovery rates (FDRs). (**C**) Performance comparison between BLASTN (left), SpacePHARER using the weighted lowest common ancestor procedure (LCA, right) at FDR = 0.02, evaluated by the number of correct (blue) and incorrect (red) predictions, for all the host predictions made at each taxonomic rank or below. BLASTN hits with >95% sequence identity and query coverage (up to 2 mismatches) were retained.

*Algorithm.* SpacePHARER is divided into five steps (Fig. 1A, Supplementary Materials). (0) Preprocess input: scan the phage genome and CRISPR spacers in six reading frames, extract and translate all putative coding fragments of at least 27 nt, with user-definable translation tables. Each query set *Q* consists of the translated ORFs *q* of CRISPR spacers extracted from one prokaryotic genome, and each target set *T* comprises the putative protein sequences *t* from a single phage. We refer to similar *q* and *t* as *hit*, and an identified host-phage relationship *Q—T* as *match*. (1) Search all *q’*s against all *t’*s using the fast, sensitive MMseqs2 protein search (Steinegger and Söding, 2017), with VTML40 substitution matrix (Müller *et al.*, 2002), gap open cost of 16 and extension cost of 2 (Supplementary Fig. S1). We optimized a short, spaced k-mer pattern for the prefilter stage (10111011) with six informative (‘1’) positions. In addition, align all *q—t* hits reported in the previous search on nucleotide level and prioritize near-perfect nucleotide hits (Supplementary Materials). (2) For each *q—T* pair, compute the *P*-value for the best hit $p_{\text{bh}}$ from first-order statistics. (3) Compute a combined score $S_{\text{comb}}$ from best-hit *P*-values of multiple hits between *Q* and *T* using a modified truncated-product method (Supplementary Materials). (4) Compute the false discovery rate (FDR = FP/(TP + FP)) and only retain matches with FDR < 0.05. For that purpose, SpacePHARER is run on a null model database and the fraction of null matches with $S_{\text{comb}}$ below a cutoff (empirical *P*-value) is used to estimate the FDR. (5) Scan 10 nt upstream and downstream of the phage’s protospacer for a possible PAM.

*Output* is a tab-separated text file. Each host-phage match spans two or more lines. The first starts with ‘#’: prokaryote accession, phage accession, $S_{\text{comb}}$, number of hits in the match. Each following line describes an individual hit: spacer accession, phage accession, $p_{\text{bh}}$, spacer start and end, phage start and end, possible 5’ PAM—3’ PAM, possible 5’ PAM—3’ PAM on the reverse strand. If requested, the spacer–phage sequence alignments are included. If taxonomic labels are provided, taxonomic reports based on the weighted lowest common ancestor (LCA) procedure described in Mirdita *et al.* (2021) are created for host LCAs of each phage genome or phage LCAs of each spacer as additional tab-separated text files.

## 3 Results

*Datasets.* We split a previously published spacer dataset (Shmakov *et al.*, 2017) of 363 460 unique spacers from 30 389 prokaryotic genomes randomly into an optimization set (20%, 6067 genomes) and a test set (80%, 24 322 genomes). The performance of SpacePHARER was evaluated on the spacer test set against a target database of 7824 phage genomes. We used two null databases: 11 304 eukaryotic viral genomes and the inverted translated sequences of the target database. Viral genomes were downloaded from GenBank in 09/2018. The performance of SpacePHARER in Figure 1C was evaluated on a validation dataset of spacers from 1066 bacterial genomes against 809 phage genomes with annotated host taxonomy (Edwards *et al.*, 2016). For each phage, we predicted the host based on the host LCA.

*Prediction quality.* At FDR = 0.05, SpacePHARER predicted 3 to 4 times more prokaryote-phage matches than BLASTN (Fig. 1B, Supplementary Fig. S2). SpacePHARER predicted the correct host for more phages than BLASTN at all taxonomic ranks, while including most of the BLASTN predictions, at better precision (Fig. 1C, Supplementary Figs S3 and S4). If the host or a close relative of a phage is absent in the database (either because the host is unidentified or the host lacks a CRISPR-Cas system), the predicted host may be correct only at a higher rank than species.

*Run time.* SpacePHARER took 12 min to process the test dataset on 2 × 6-core Intel E5-2620v3 CPUs, 47 times faster than BLASTN (575 min).

## 4 Conclusion

SpacePHARER is 1.4 to 4× more sensitive than BLASTN in detecting phage–host pairs, due to searching with protein sequences, optimizing short sequence comparisons, and combining statistical evidence, and it is fast enough to analyze large-scale genomic and metagenomic datasets.

## Funding

E.L.K. is a FEBS long-term fellowship recipient and an EMBO non-stipendiary long-term fellow. The work was supported by the ERC’s Horizon 2020 Framework Programme [‘Virus-X’, project no. 685778] and the BMBF CompLifeSci project horizontal4meta. 

*Conflict of Interest*: none declared. 

## Data availability

The data used to benchmark SpacePHARER and BLASTN are publicly available from ftp://ftp.ncbi.nih.gov/pub/wolf/_suppl/spacerome/ and http://edwards.sdsu.edu/PhageHosts/. The viral genomes were downloaded from NCBI Genbank in 09/2018.

## Supplementary Material

btab222_Supplementary_Data
