# Supplementary material for: SpacePHARER: sensitive identification of phages from CRISPR spacers in prokaryotic hosts
Source: Bioinformatics. 2021 Apr 1;37(19):3364–6. doi: 10.1093/bioinformatics/btab222 (PMC8504623; doi:10.1093/bioinformatics/btab222)
Supplement: btab222_Supplementary_Data [file btab222_supplementary_data.pdf]

Supplementary Material for SpacePHARER: Sensitive identification of phages from CRISPR spacers in prokaryotic hosts

Zhang R.,[1] Mirdita M.,[1] Levy Karin E.,[1] Norroy C.,[1] Galiez C.,[1,2] and Söding J.[1,3]

[1] Quantitative and Computational Biology, Max Planck Institute for Biophysical Chemistry, Göttingen, Germany
[2] Univ. Grenoble Alpes, CNRS, Grenoble INP/Institute of Engineering Univ. Grenoble Alpes, Grenoble, France
[3] Campus-Institut Data Science (CIDAS), Göttingen, Germany.

I. ALGORITHM DESCRIPTION

The query spacer set Q has N_q translated ORFs q of CRISPR spacers ($Q = \{q_1, ...q_{N_q}\}$) from one prokaryotic genome. Phage proteome target set T has N_t phage protein sequences t ($T = \{t_1, ...t_{N_t}\}$). These protein sequences are extracted in the input preprocessing step (Step 0) of the algorithm from each spacer set and each phage genome by scanning them in six translational frames. We refer to similarity between q and t as hit, and similarity between Q and T as match. The SpacePHARER algorithm relies on a statistic for the combination of hits between a spacer sequence set and a phage protein sequence set. The idea is that combining together several sub-significant hits (due to weak homologies or the typical length of spacers) can be highly informative and result in a significant match. Steps 2 and 3 of the algorithm test if the pairwise P-values of the best hit of sequences in the query set with those in the target set are due to homologous relationships or entirely due to chance.

A. (1) MMseqs2 protein-level search

The SpacePHARER algorithm first searches all q's against all t's using the fast, sensitive MMseqs2 protein-level search [10], with VTML40 substitution matrix [8], gap open cost of 16, gap extension cost of 2, and a short, spaced k-mer pattern for the prefilter stage (10111011) with six informative ('1') positions. Spaced k-mers are utilized in MMseqs2 to reduce the correlation between k-mers at neighboring positions, and to achieve better sensitivity and speed. The spaced k-mer pattern is chosen such that it is short in length in order to produce consecutive double k-mer matches (which are demanded by MMseqs2) within spacer fragments of 10-12 aa, and that the number of maximum overlapping informative positions is minimized.

Perfect or near-perfect hits (with no or 1-2 mismatches on the nucleotide level) are shown to be very reliable signals in predicting phage-host relationship and improve the taxonomic certainty of the prediction, even if there is only a single hit between a phage-host pair [4]. However, those hits are not well reflected in the pairwise P-value of the protein-level search. Therefore, all $q - t$ hits reported from the sensitive protein-level search will be aligned again on the nucleotide level with match reward of 1, mismatch penalty of 1, gap open cost of 10 and gap extension cost of 2. The protein-level search will compute a protein pairwise P-value (p_{prot}) for each hit and nucleotide alignment a nucleotide pairwise P-value (p_{nucl}). In order to prioritize near-perfect hits on the nucleotide level to gain precision without losing much sensitivity, we compute the pairwise P-value as

$$\exp\left(\min\left\{\left(0.5\log p_{\text{prot}} + 0.5\log p_{\text{nucl}}\right), \log p_{\text{nucl}}\right\}\right) \quad (1)$$

B. (2) Computing P-value of best hit

All hits of each q against the N_t proteins in a specific phage genome T are examined by their pairwise P-values, and the hit with the lowest pairwise P-value ("best hit") is retained. SpacePHARER computes the P-value of the best hit $p_{\text{bh}}(q)$ using first order statistics, i.e. the P-value of taking the minimum pairwise P-value ($p(q)$), given that a total of N_t pairwise P-values were examined:

$$p_{\text{bh}}(q) = P\left(p\left(q\right) \leq p\right) = 1 - \left(1 - p\right)^{N_t} \quad (2)$$

C. (3) Combining P-values using a modified truncated product method

In this step, we aim to combine the evidence from several best hits between a spacer set Q and a phage genome T. We sort the p_{bh} of the given set Q of N_q sequences in ascending order and denote the i'th p_{bh} as p_i. When combining independent P-values of individual hits, one needs to take into account the number of individual hits and the strength of each hit. The truncated product method combines independent P-values into a score by multiplying all $p_{\text{bh}}(q)$ smaller than a threshold p_0 [14],

$$S_{\text{comb}} = -\log \prod_{i=1}^{N_q} p_i^{I(p_i < p_0)}, \quad (3)$$

where $I(\cdot)$ is the indicator function that returns 1 if the argument is true and otherwise returns 0.

In SpacePHARER, we modified the truncated product method for better performance. We take the product of the smallest best-hit P-value p_1 times the ratio between

p_i and the threshold p_0 for all further p_i below the threshold p_0:

$$S_{\text{comb}} = -\log \left(p_1 \times \prod_{i=2}^{N_q} \left(\frac{p_i}{p_0} \right)^{I(p_i < p_0)} \right) \quad (4)$$

For the threshold, we set $p_0 = 1/(N_q + 1)$, which corresponds to marginal significance, with an E-value of $N_q/(N_q + 1)$ just below 1. This ensures that the combined score for null model-distributed P-values p_i only rarely gets boosted by a contribution from the second-best p_i.

D. (4) Determining true predictions

SpacePHARER predicts matches *de novo*, i.e. without relying on any known phage-host relationships, by controlling for estimated false discovery rate (FDR). The FDR is the proportion of false predictions among all predictions:

$$\text{FDR} = \frac{\text{FP}}{\text{FP} + \text{TP}} \quad (5)$$

We implemented an FDR estimation approach similar to that of the R package "fdrtool" [11]. In essence, we estimate the FDR by a Grenander decreasing density estimate of the empirical cumulative distribution function (ECDF). This non-parametric approach achieves its robustness by ensuring monotonicity of the FDR.

SpacePHARER uses a null model dataset to estimate the proportion of false predictions. The same search and statistical computation procedures described in Steps 1, 2 and 3 of the algorithm are performed on a given null model dataset, e.g. inverted phage ORFs or eukaryotic viral ORFs. Inverting target ORFs as null model dataset can be easily performed by specifying one parameter when preparing the input.

To compute an empirical P-value for each query spacer set Q, we sort for each Q the combined scores S_{comb} of matches in the original target dataset of phage proteomes in ascending order. For each S_{comb} value in the target dataset, we calculate an empirical P-value p_{emp} by using the fraction of $Q-T$ matches with a combined score that is below S_{comb} in the null model dataset. We denote the number of $Q-T$ matches below the cutoff as K and the total number of matches using the null model dataset as N_{null}. The empirical P-value is then computed as

$$p_{\text{emp}}(S_{\text{comb}}) = \frac{K + 0.5}{N_{\text{null}} + 1}, \quad (6)$$

where, to stabilize the estimate, we used half pseudocounts with P-values at 0 and 1. In the following, we abbreviate these empirical P-values as p, or p_Q for query set Q.

If we knew the fraction π_0 of false positives among all $Q-T$ matches, we could in principle estimate the false discovery rate simply as

$$\text{FDR}(p) = \frac{\text{FP}_p}{(\text{TP} + \text{FP})_p} \approx \frac{p\,\pi_0}{F_{\text{emp}}(p)}, \quad (7)$$

where $p\,\pi_0$ is the fraction of false positives with empirical P-value less than p_i. $F_{\text{emp}}(p)$ is the empirical cumulative distribution function of the p_Q, in other words $F_{\text{emp}}(p)$ is the number of query sets Q with best matches $p_Q \leq p$.

We can increase the robustness of the estimate by using the fact that the true probability distribution of P-values $f(p)$ must be monotonously decreasing. This will also ensure that the FDR decreases with increasing p, which is often violated with the simple procedure above. The Grenander estimate [11] is a simple, efficient procedure to obtain a robust estimate $\hat{F}(p)$ of $F(p)$ from $F_{\text{emp}}(p)$ that has monotonously decreasing density $\hat{f}(p) = d\hat{F}(p)/dp$. We simply obtain the convex hull of the area under the $F_{\text{emp}}(p)$ curve, that is, the smallest function $\hat{F}(p)$ with $\hat{F}(p) \geq F_{\text{emp}}(p)$ that yields a convex area under the curve. This results in a piecewise constant, monotonously decreasing density function $\hat{f}(p) = d\hat{F}(p)/dp$ with steps at points p_i with $p_{\text{last}} = 1$. We estimate the proportion of true null hypotheses π_0 as the average density using the last two steps,

$$\pi_0 = \frac{\hat{F}(p_{\text{last}}) - \hat{F}(p_{\text{last}-2})}{p_{\text{last}} - p_{\text{last}-2}}. \quad (8)$$

Finally, we compute the estimated FDR corresponding to each empirical P-value p (Fig.1A) as

$$\text{FDR}(p) = \frac{\text{FP}_p}{(\text{TP} + \text{FP})_p} = \frac{p\,\pi_0}{\hat{F}(p)}. \quad (9)$$

By default, SpacePHARER has an FDR cutoff of 0.05, and reports all matches in the test whose S_{comb} corresponds to this FDR value or lower. Users can select other suitable FDR cutoffs to retain more or fewer predictions.

E. (5) Scanning for possible PAMs

For some CRISPR-Cas systems, protospacer adjacent motifs (PAMs) are required for the recognition of foreign invader sequences. After reporting phage-host pairs and their hits, SpacePHARER can perform a scan for possible PAMs. For this, SpacePHARER by default extracts 10 nt long fragments flanking the matched protospacer region at the 5' and 3' side, in guide-centric orientation (PAM is located on the strand that matches the spacer sequence). Users can increase or decrease the length of the flanking sequence. Both the 5' and 3' flanking sequences are searched in a list of consensus PAM patterns from representative CRISPR-Cas systems [5]. Since many CRISPR detection tools cannot reliably predict the orientation of

the CRISPR array, the 5' and 3' flanking sequences on the reverse strand are also searched and two additional possible PAMs are reported. Users should refer to all possible PAMs without the accurate orientation information of the array.

II. OPTIMIZING PARAMETERS FOR SHORT FRAGMENTS SEARCH

Different substitution matrices are optimal for comparing sequences that have diverged to different degrees. By default, MMseqs2 search [10] uses the BLOSUM62 matrix with standard gap penalties: gap open cost of 11 and gap extend cost of 1 , which is more suited for long alignments and detecting weak protein similarities. Conversely for shorter sequences and higher protein similarity, one should consider a "shallower" (higher bit score per aligned column) matrix, and higher gap penalties to prevent gaps [9]. Searching with VTML40 matrix [8] with gap open cost of 16 and gap extend cost of 2 yielded the highest sensitivity with 20% on our test dataset at FDR cutoff of 0.05 (Figure S2). We introduced a series of VTML matrices in MMseqs2 to solve general problems of short sequence search. After introducing the additional nucleotide alignment step, the search parameter combination (VTML40 matrix, gap open cost of 16 and gap extend cost of 2) remains the highest in sensitivity (result not shown).

III. PREDICTING MATCHES USING BLASTN

We compared SpacePHARER's performance with the state-of-the-art method using BLASTN. To generate a comparable result, we performed the search step with BLASTN and the downstream FDR control with SpacePHARER. We used BLASTN [1] to first query the 80% test spacer dataset against 7,824 phage genomes, then against 7,824 inverted phage genomes or 11,304 eukaryotic viral genomes as a null model database. For all searches we used the parameters: -max_target_seqs 10000000 -dust no -word_size 7 -outfmt '6 std qcovs' and recorded the running time. Hits with at least 95% sequence identity and 95% query(spacer) coverage (i.e., one or two mismatches were allowed) were retained. We grouped the hits into matches (unique phage-host genome pairs) and retained the minimum pairwise E-value of the hits. We sorted the pairwise E-values of hits in ascending order for both searches and counted the matches at a given pairwise E-value cutoff. Therefore, we could calculate an FDR in the same way SpacePHARER does (described in section I.D) and compare the number of true predictions produced by the two methods (Figure 1B).

At FDR = 0.05, SpacePHARER predicted 2 and 1.5× more matches than BLASTN using 90% and 85% sequence identity and query coverage cutoffs (i.e allowing up to 4 and 6 mismatches, respectively) (Figure S2).

IV. HOST TAXONOMIC RANK ANALYSIS

To assess the sensitivity of SpacePHARER at different host taxonomic rank, we searched with CRISPR spacers extracted from 1,066 bacterial genomes against 809 phage genomes with annotated host taxonomy [4], then against inverted ORFs of the 809 phage genomes as null model dataset. For each phage, SpacePHARER predicted the host's lowest common ancestor (LCA) based on a weighted LCA procedure [7].

We demanded a stricter FDR cutoff of 0.02 for matches that should be taken into account for the host taxonomic rank prediction. In order to limit the number of false taxonomic predictions due to incomplete databases, the LCA result was further corrected according to the average nucleotide sequence identity of the reported matches [6]. We used the following cutoffs for maximal taxonomic resolution: $> 86\%$ (species), $> 84\%$ (genus), $> 82\%$ (family), $> 80\%$ (order), $> 78\%$ (class), $> 76\%$ (phylum), $> 74\%$ (kingdom). Lower values were assigned at the superkingdom level. The taxonomic FDR cutoff and sequence identity cutoffs are user-definable parameters for the weighted LCA procedure.

We searched with the above-mentioned spacer dataset against phage genomes using BLASTN with parameters: blastn-short -dust no -word_size 7 -outfmt '6 std qcovs' -evalue 1 -gapopen 10 -gapextend 2 -penalty -1 [4]. Hits with at least 95% sequence identity and 95% query(spacer) coverage were retained (i.e., one or two mismatches were allowed). For each phage, the bacterium with the lowest pairwise E-value was predicted to be its host. Note that in Edwards et al., the authors searched with the phage genomes against the spacer dataset, and demanded 100% spacer coverage.

For ranks lower than phylum, we only included the predictions with the taxonomic resolution of the respective rank or below. At the species level, SpacePHARER predicted 142/237 hosts (60%), compared to 112/232 hosts of BLASTN (48%). SpacePHARER predicted the correct host for more phages at all taxonomic ranks, while including most of the BLASTN predictions on the same rank and sometimes even those agreeing only on a higher rank(Figure 1C, Figure S3).

Incomplete reference databases remain an issue for phage-host relationship predictions. To simulate scenarios where the database is very incomplete, we progressively exclude 25% and 50% of the host genomes in the spacer dataset, and compare the performance between BLASTN and SpacePHARER. SpacePHARER predicted the correct host for more phages than BLASTN at all taxonomic ranks when we searched with 50% and 75% of original host spacer dataset (Figure S4).

V. IDENTIFYING MIS-ANNOTATIONS IN EUKARYOTIC VIRAL DATASET

Throughout this study we used the set of *eukaryotic viral genomes* as a null model dataset, assuming any match between a prokaryotic genome and a eukaryotic virus is false. Here, we used SpacePHARER's second mode of FDR control to detect viruses that were potentially mis-annotated as eukaryotic viruses. To that end, we first ran the SpacePHARER workflow with the full spacer dataset against the *eukaryotic viral genomes* as the target database, and then, against inverted *eukaryotic viral* ORFs as the null model database. We used the null set to estimate the FDR as described in section I.D.

By applying the same FDR cutoff of 0.05, we identified 11 viruses out of the 11,304 that matched a prokaryotic host (yielding a total of 12 matches). We observed three groups within these matches. The first group consisted of two matches between the smacovirus family (KP264966.1 and KY086299.1) and the archaeon CP005934.1 (*Candidatus Methanomassiliicoccus intestinalis*). Indeed this family has been recently reported as mis-annotated as "eukaryotic virus" by Díez-Villaseñor and Rodriguez-Valera [3]. The second group consisted of two matches between KT809302.1 (Haloarcula californiae icosahedral virus 1) and family *Halobacteriaceae* (CP001687.1 and LIST01000008.1). These matches are likely due to mis-annotation of the virus as "eukaryotic virus". The labeled host of this virus is *Haloarcula californiae*, which is an archaeon that belongs to the same family as our matches. The third group consisted of 8 members of the genus *Mimivirus* that were matched to HE978663.1 (*Ruminococcus sp.* JC304) and JAAF01000022.1 (*Fusobacterium necrophorum* DAB). Table I shows the standard output from SpacePHARER of this search. We suspect the matches of the third group are due to spacer mis-annotation and do not represent a real virus-host relationship. It was previously reported that Mimiviruses acquire bacterial genes, even of the class *Clostridia* [12][13]. In the case of *Ruminococcus sp.* JC304, when we inspected the bacterial genomic region from which the spacers were extracted, we found that the entire region is likely to be a full bacterial ORF, rather than a CRISPR array. Thus, we conclude that in these cases, the mis-annotation is of the CRISPR array, rather than of the virus.

VI. SOFTWARE VERSIONS

Name	Version
SpacePHARER	Git: 1d1f1b2
BLASTN	2.9.0+

TABLE II. Software versions used in this manuscript.

[1] Altschul, S.F. et al (1990). Basic local alignment search tool. *J. Mol. Biol.*, **215**(3), 403–410.

[2] Brunson, J.C. (2020). ggalluvial: Layered grammar for alluvial plots. *J. Open Source Softw.*, **5**(49), 2017.

[3] Díez-Villaseñor, C. and Rodriguez-Valera, F. (2019). CRISPR analysis suggests that small circular single-stranded dna smacoviruses infect archaea instead of humans. *Nat. Commun.*, **10**(1), 294.

[4] Edwards, R.A. et al (2015). Computational approaches to predict bacteriophage–host relationships. *FEMS Microbiol. Rev.*, **40**(2), 258–272.

[5] Leenay, R.T. and Beisel, C.L. (2017). Deciphering, communicating, and engineering the crispr pam. *Journal of molecular biology*, **429**(2), 177–191.

[6] Levy Karin, E. et al (2020). Metaeuk—sensitive, high-throughput gene discovery, and annotation for large-scale eukaryotic metagenomics. *Microbiome*, **8**(1), 48.

[7] Mirdita, M. et al (2021). Fast and sensitive taxonomic assignment to metagenomic contigs. *Bioinformatics.* btab184.

[8] Müller, T. et al (2002). Estimating amino acid substitution models: A comparison of Dayhoff's estimator, the resolvent approach and a maximum likelihood method. *Mol. Biol. Evol.*, **19**(1), 8–13.

[9] Pearson, W.R. (2013). Selecting the Right Similarity-Scoring Matrix. *Current Protocols in Bioinformatics*, **43**(1), 3.5.1–3.5.9.

[10] Steinegger, M. and Söding, J. (2017). MMseqs2 enables sensitive protein sequence searching for the analysis of massive data sets. *Nat. Biotechnol.*, **35**(11), 1026–1028.

[11] Strimmer, K. (2008). A unified approach to false discovery rate estimation. *BMC Bioinformatics*, **9**(1), 303.

[12] Yoshida, T. et al (2011). Mimivirus reveals mre11/rad50 fusion proteins with a sporadic distribution in eukaryotes, bacteria, viruses and plasmids. *Virology journal*, **8**, 427–427.

[13] Yutin, N. et al (2014). Origin of giant viruses from smaller dna viruses not from a fourth domain of cellular life. *Virology*, **466-467**, 38 – 52. Special issue: Giant Viruses.

[14] Zaykin, D. et al (2002). Truncated product method for combining p-values. *Genet. Epidemiol.*, **22**(2), 170–185.

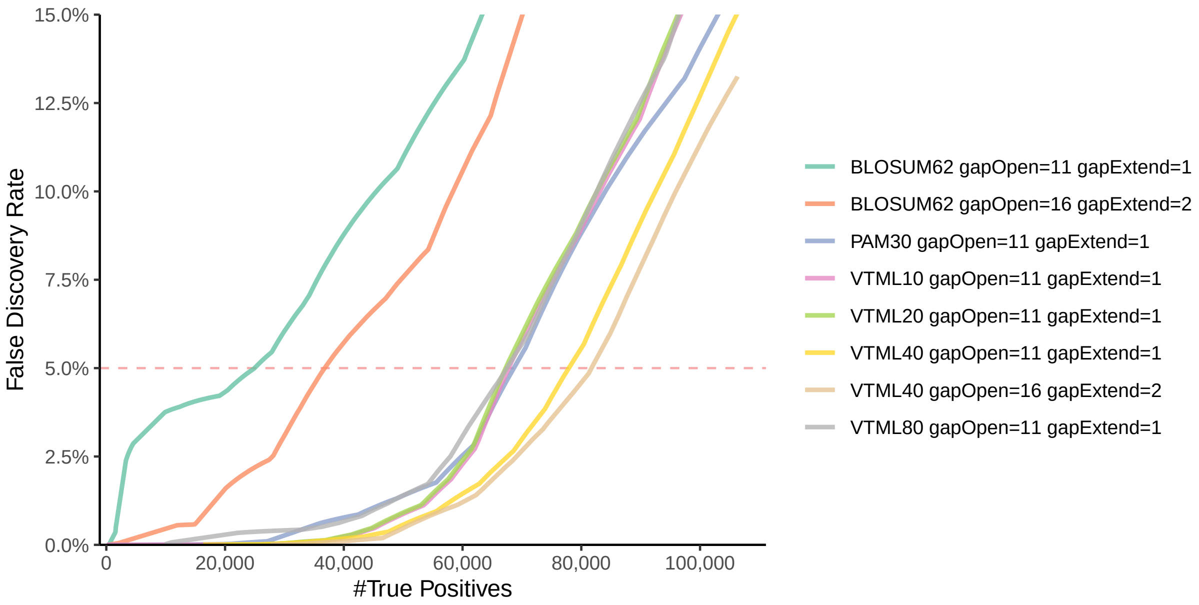

FIG. 1. Performance comparison of SpacePHARER with different search parameters (substitution matrix and gap penalties), evaluated by the number of true positive (TP) predictions at different false discovery rates (FDRs). Predictions were made by using an optimization spacer dataset (6,067 genomes, 20% of all prokaryotic genomes) against a database of 7,824 phage genomes, with inverted phage ORFs as null model database. Searching with VTML40 matrix with gap open (16) and gap extend (2), among various combinations of substitution matrix and gap penalties, yields more true positive matches than any other parameter combination at FDR cutoff of 0.05.

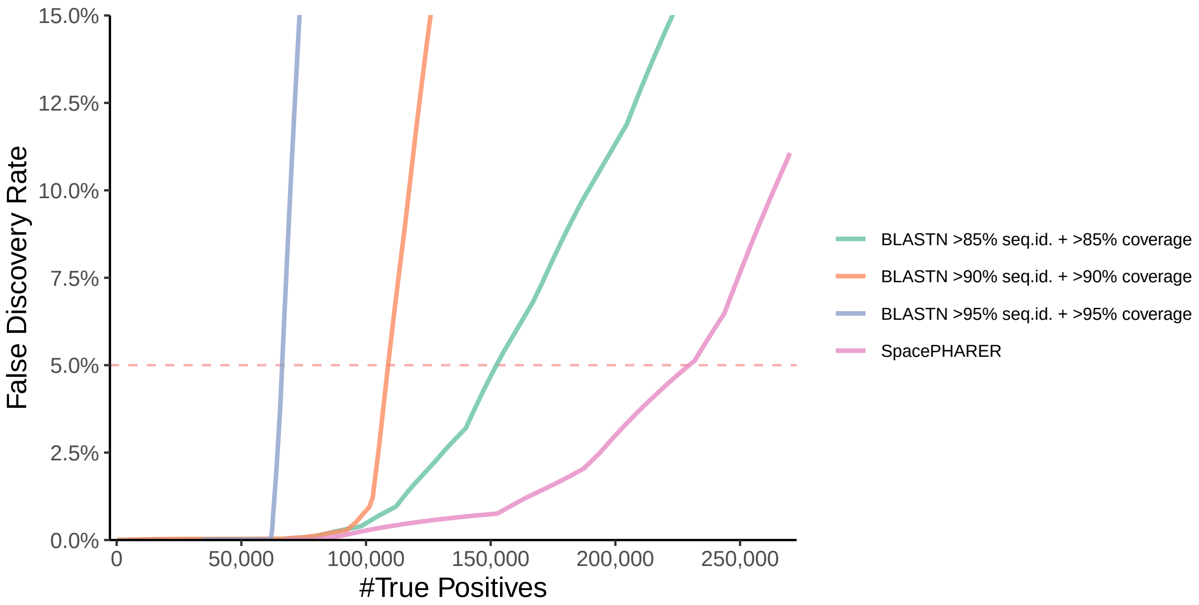

FIG. 2. Performance comparison of SpacePHARER with BLASTN using different sequence identity and query coverage cutoffs (95%, 90% and 85%), evaluated by the number of true positive (TP) predictions at different false discovery rates (FDRs). Predictions were made by using a spacer test dataset (24,322 genomes, 80% of all prokaryotic genomes) against a database of 7,824 phage genomes, with inverted phage ORFs as null model database. (Note that the FDR control procedure developed for SpacePHARER is not standard for BLASTN and has been applied here only for the purpose of FDR analysis.)

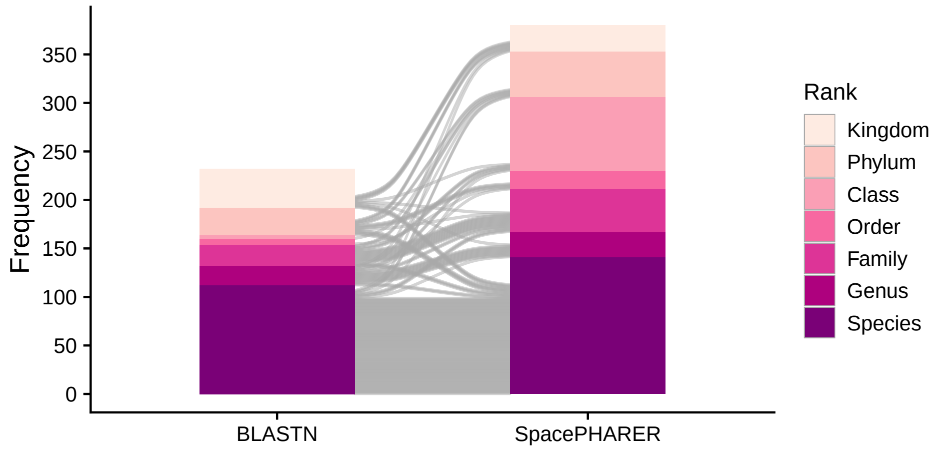

FIG. 3. Performance comparison of BLASTN (left) and SpacePHARER (right), evaluated by the number of host predictions that agree with annotated host taxonomy at different taxonomic ranks. The grey alluvia [2] represent the host predictions that were made by both SpacePHARER and BLASTN. Predictions were made using a validation spacer dataset (1,066 genomes) against a validation database of 809 phage genomes with annotated host taxonomy. SpacePHARER prediction was further corrected with inverted phage ORFs as null model database, and FDR cutoff of 0.02.

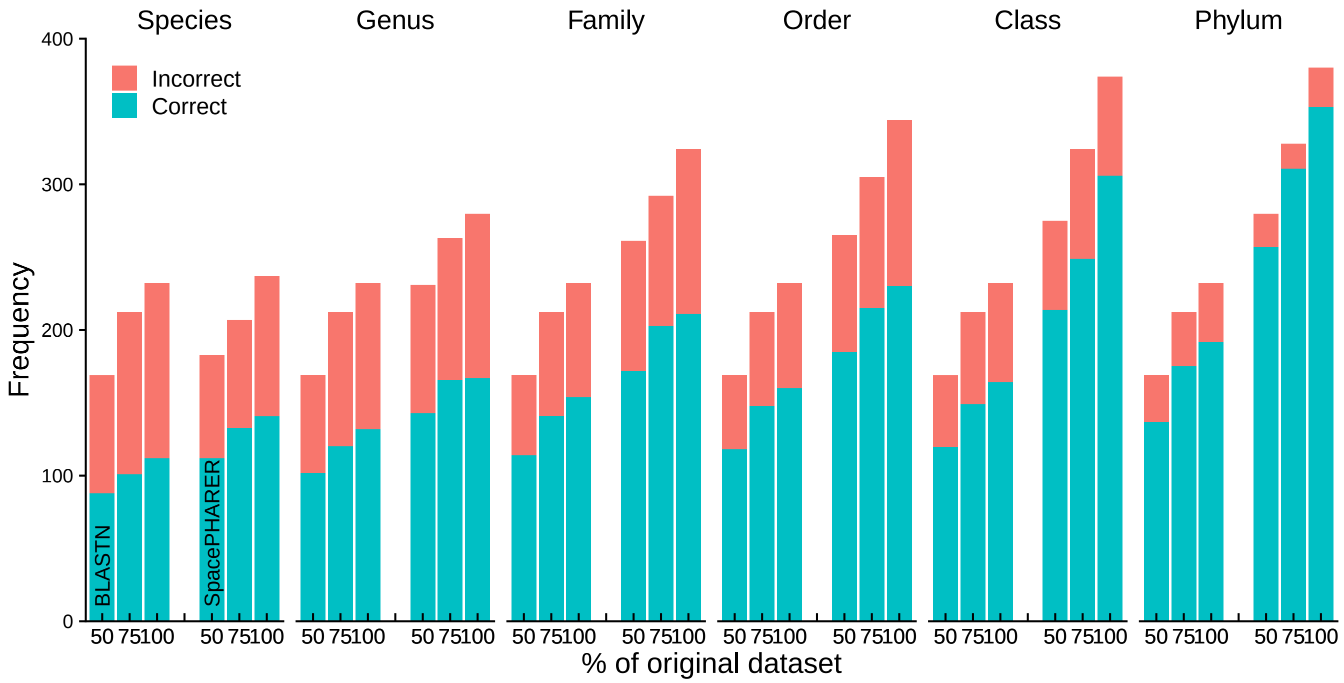

FIG. 4. Performance comparison of BLASTN (left) and SpacePHARER (right) as for Figure 1C, but on incomplete databases. The host spacer dataset was progressively depleted from 100% of genomes (1,066) to 75% (800) and 50% (533). Performance is evaluated by the number of host predictions that agree with annotated host taxonomy at different taxonomic ranks.

#CP005934.fas	KP264966.1	7.588E+01	6					
>CP005934.1_930280_937725_19_spacer_931524_35	KP264966.1	1.023E-04	35	3	546	578	CCT|-	-|AGG
>CP005934.1_930280_937725_20_spacer_931590_37	KP264966.1	2.833E-04	1	36	2241	2206	CCT|-	-|AGG
>CP005934.1_930280_937725_23_spacer_931792_37	KP264966.1	1.034E-09	1	36	1821	1786	CCA|-	-|TGG
>CP005934.1_930280_937725_24_spacer_931860_37	KP264966.1	3.121E-07	3	35	606	638	CCA|TGG	-|TGG
>CP005934.1_930280_937725_25_spacer_931928_36	KP264966.1	3.399E-13	1	36	2161	2126	CCG|-	-|CGG
>CP005934.1_930280_937725_25_spacer_931928_36	KP264966.1	1.713E-11	2	34	2160	2128	CCG|-	-|CGG
#CP005934.fas	KY086299.1	5.640E+01	4					
>CP005934.1_930280_937725_19_spacer_931524_35	KY086299.1	6.205E-04	35	3	1922	1890	CCT|-	-|AGG
>CP005934.1_930280_937725_23_spacer_931792_37	KY086299.1	3.399E-13	2	37	641	676	CCA|-	-|AGG
>CP005934.1_930280_937725_20_spacer_931590_37	KY086299.1	4.613E-05	1	36	220	255	CCT|-	-|TGG
>CP005934.1_930280_937725_23_spacer_931792_37	KY086299.1	3.399E-13	1	36	640	675	CCA|-	-|TGG
#LIST01000008.fas	KT809302.1	1.295E+01	1					
>LIST01000008.1_120573_126312_45_spacer_123484_36	KT809302.1	2.376E-06	36	1	22375	22340	-|-	TTC|-
#CP001687.fas	KT809302.1	2.639E+01	2					
>CP001687.1_1415738_1419119_25_spacer_1417344_34	KT809302.1	1.137E-07	6	32	6826	6852	-|CAAGAA	-|ACGGGATT
>CP001687.1_1415738_1419119_25_spacer_1417344_34	KT809302.1	1.137E-07	32	6	6852	6826	-|CAAGAA	-|ACGGGATT
#HE978663.fas	JN258408.1	1.054E+02	2					
>HE978663.1_7481_7851_2_spacer_7588_70	JN258408.1	1.755E-28	2	70	806538	806606	TTC|-	-|-
>HE978663.1_7481_7851_4_spacer_7765_58	JN258408.1	5.707E-20	2	58	806715	806771	TTC|-	-|-
#HE978663.fas	JX885207.1	9.775E+01	2					
>HE978663.1_7481_7851_2_spacer_7588_70	JX885207.1	1.755E-28	2	70	767273	767341	TTC|-	-|-
>HE978663.1_7481_7851_4_spacer_7765_58	JX885207.1	1.187E-16	2	58	767450	767506	TTC|-	-|-
#HE978663.fas	KF527229.1	8.186E+01	2					
>HE978663.1_7481_7851_1_spacer_7510_49	KF527229.1	2.905E-18	2	49	935992	935945	TTC|-	-|-
>HE978663.1_7481_7851_4_spacer_7765_58	KF527229.1	5.707E-20	2	58	935836	935780	TTC|-	-|-
#HE978663.fas	KU877344.1	9.775E+01	2					
>HE978663.1_7481_7851_2_spacer_7588_70	KU877344.1	1.755E-28	2	70	780352	780420	TTC|-	-|-
>HE978663.1_7481_7851_4_spacer_7765_58	KU877344.1	1.187E-16	2	58	780529	780585	TTC|-	-|-
#HE978663.fas	JX975216.1	1.015E+02	2					
>HE978663.1_7481_7851_2_spacer_7588_70	JX975216.1	1.755E-28	2	70	781866	781934	TTC|-	-|-
>HE978663.1_7481_7851_4_spacer_7765_58	JX975216.1	2.682E-18	2	58	782043	782099	TTC|-	-|-
#HE978663.fas	MG779360.1	1.093E+02	2					
>HE978663.1_7481_7851_2_spacer_7588_70	MG779360.1	3.447E-30	2	70	9786	9854	-|-	-|-
>HE978663.1_7481_7851_4_spacer_7765_58	MG779360.1	5.707E-20	2	58	9963	10019	-|-	-|-
#HE978663.fas	JN885991.1	4.536E+01	2					
>HE978663.1_7481_7851_3_spacer_7687_49	JN885991.1	2.061E-02	2	46	497977	498021	CCT|-	TTG|AGG
>HE978663.1_7481_7851_4_spacer_7765_58	JN885991.1	5.707E-20	2	58	498055	498111	-|-	-|-
#JAAF01000022.fas	KY684109.1	1.295E+01	1					
>JAAF01000022.1_41_3914_26_spacer_1726_36	KY684109.1	2.376E-06	1	36	185359	185324	TCT|TGAAGTTT	TCA|-

TABLE I. Sample output format of SpacePHARER, demonstrated by matches when searching the full spacer dataset against eukaryotic viral ORFs as a target database and inverted eukaryotic viral ORFs as null model database. Each match line starts with '#', followed by the prokaryote accession (the file from which spacers were extracted), viral genome accession, S_{comb} and the number of hits in the match. Each hit line starts with '>', followed by the spacer sequence header, viral genome accession, p_{bh}, spacer start, spacer end, viral genome start, viral genome end, and the possible PAM sequences on forward and reverse strand (5'|3'). Additionally (not shown), the aligned sequences can be printed following each hit line.
